# K-Means Clustering Reveals Long-Term Thyrotropin Receptor Antibody Patterns in Graves’ Disease: Insights from a 10-Year Study with Implications for Graves’ Orbitopathy

**DOI:** 10.3390/jcm14051734

**Published:** 2025-03-04

**Authors:** Jungyul Park, Jae Hyun Kim, Hee-young Choi, Jinmi Kim, Sang Soo Kim, Suk-woo Yang

**Affiliations:** 1 Department of Ophthalmology, Seoul St. Mary’s Hospital, College of Medicine, The Catholic University of Korea, Seoul 06591, Republic of Korea; ophjyp@naver.com; 2 Department of Ophthalmology, School of Medicine, Pusan National University Hospital, Busan 49241, Republic of Korea; ot2018175@naver.com; 3 Department of Biostatistics, Clinical Trial Center, Biomedical Research Institute, Pusan National University Hospital, Busan 49241, Republic of Korea; jinmi@pusan.ac.kr; 4 Department of Endocrinology, School of Medicine, Pusan National University Hospital, Busan 49241, Republic of Korea; drsskim7@gmail.com

**Keywords:** thyrotropin receptor antibody (TRAb), Graves’ disease, K-means clustering, longitudinal patterns, Graves’ orbitopathy

## Abstract

**Background/Objectives**: We aimed to explore long-term trajectories of thyroid-stimulating hormone receptor antibody (TRAb) in patients with Graves’ disease (GD) and to identify key factors associated with TRAb normalization. We also investigated whether these trajectories correlate with Graves’ orbitopathy (GO) comorbidity. **Methods**: We retrospectively reviewed 403 patients with GD who had an initial TRAb level ≥ 1.5 IU/L between 2010 and 2021, monitoring their TRAb levels for at least 3 years. K-means clustering was performed to categorize patients into distinct TRAb change patterns (A, B, C, D). We employed a Cox regression–based time-to-event model, expressing results as “Survival ratio” rather than the conventional Hazard ratio, to reflect the proportion of patients achieving TRAb normalization over time. Key variables included age, sex, initial TRAb, and GO comorbidity. **Results**: Four unique TRAb patterns emerged, differing primarily in baseline TRAb levels, duration of GD, and treatment approaches. Pattern A demonstrated the highest TRAb normalization rate (96%), whereas Patterns B (80%), C (29%), and D (13%) showed lower probabilities. Regrouping into A vs. BCD further emphasized the distinct normalization profile of Pattern A. A higher “Survival ratio” was observed in female patients and those with baseline TRAb < 6.14 IU/L. In contrast, patients whose TRAb levels were ≥6.14 IU/L frequently exhibited persistently elevated values over a decade. GO comorbidity did not significantly differ among the four patterns. **Conclusions**: K-means clustering revealed four unique TRAb change patterns in GD, with baseline TRAb (stratified by the median of 6.14 IU/L) and sex emerging as significant predictors of normalization. These findings highlight the importance of early TRAb monitoring and tailored therapeutic strategies, particularly for those with persistently elevated TRAb levels.

## 1. Introduction

Graves’ disease (GD) is an autoimmune disorder primarily characterized by excessive thyroid hormone secretion. Thyroid-stimulating hormone receptor antibodies (TRAb) play a key etiological role in this process by stimulating thyroid-stimulating hormone (TSH) receptors, thereby leading to hyperthyroidism [1]. The TRAb assay is a vital tool for diagnosing GD owing to its high sensitivity and specificity [2]. In addition, TRAb levels typically decline as GD is managed with antithyroid medications or thyroidectomy, although radioactive iodine (RAI) treatment can transiently elevate TRAb levels during the first year [2,3,4]. During the clinical course of GD treatment, the specific cutoff values of TRAb serve as good predictors of relapse [5]. By evaluating whether TRAb remains elevated or decreases to normal ranges, clinicians can make more informed decisions regarding the continuation or cessation of antithyroid therapy [6,7].

TRAb also plays an important role in the pathogenesis of Graves’ orbitopathy (GO) by directly stimulating orbital fibroblasts, leading to increased hyaluronic acid synthesis, adipogenesis, and the release of pro-inflammatory cytokines such as IL-1β and TNF-α [8,9]. This autoimmune activation contributes to orbital tissue expansion, edema, and fibrosis, which are key features of GO. Studies have consistently shown that TRAb levels correlate with GO severity, with higher titers associated with increased risk of disease progression [10,11]. Additionally, persistently elevated TRAb after treatment has been linked to a higher likelihood of GO recurrence and worsening [11]. Given its role in disease progression, high TRAb is also considered a risk factor for GO progression following RAI treatment and guides clinicians in determining whether steroid prophylaxis should be prescribed to prevent GO exacerbation after RAI treatment [12,13]. These findings highlight TRAb as not only a marker of autoimmune thyroid disease but also a potential predictor of extrathyroidal involvement, including GO development and severity.

As TRAb is an important factor in both the pathogenesis and treatment guidelines for GD and GO, investigating its long-term changes in GD is worthwhile. While several studies have explored the overall average changes in TRAb levels during different GD treatments, there is a notable research gap in classifying the distinct patterns of TRAb changes over time during GD treatment and their relationship with GO comorbidity [2,4,14]. Identifying distinct patterns of TRAb changes could provide deeper insights into disease prognosis and help optimize individualized treatment strategies.

Clinical observations suggest that TRAb levels often remain elevated beyond two years in a subset of patients despite treatment [15]. In a previous study conducted in a different cohort, we examined TSI normalization over a 3-year period and found that only 43.4% of patients achieved normalization following steroid therapy [16]. Given that TRAb and TSI trajectories are known to exhibit similar trends, we hypothesized that persistent TRAb elevation may also have long-term prognostic implications in GD. Thus, in this study, we specifically analyzed TRAb trajectories over a minimum follow-up period of 3 years to further characterize its long-term behavior and clinical relevance.

Predicting TRAb trends can also aid in clinical decision-making by guiding the duration of antithyroid medication and monitoring for extrathyroidal manifestations, including GO [17]. Therefore, the primary objective of this study was to characterize the long-term patterns of TRAb changes during GD treatment and to evaluate their association with the presence of GO. We also aimed to identify key factors related to TRAb normalization. By investigating these aspects, we hope to enhance our understanding of TRAb dynamics and their implications for the management and potential progression of GO.

## 2. Materials and Methods

### 2.1. Patient Recruitment

A retrospective analysis was conducted on the medical records of adult patients (aged 19 years and above) diagnosed with GD at Tertiary National University Hospital between the years 2010 and 2021. The inclusion criteria for the study required patients to have a TRAb level exceeding 1.5 IU/L and a minimum of 3-year follow-up on TRAb levels.

Patients were excluded if they had insufficient laboratory data, a prior diagnosis of Graves’ orbitopathy (GO)—which may have introduced confounding effects due to prior immunosuppressive treatments—or incomplete treatment records. The Institutional Review Board (IRB) of Pusan National University Hospital approved this study, as indicated by the assigned IRB number 2306-030-128.

### 2.2. Clinical and Laboratory Assessment

Serum TSH receptor autoantibodies (TRAb) were measured using a second-generation TRAK human radio-receptor assay (Thermo Fisher Scientific, Freiburg, Germany), which has a reference range of 0–1.5 IU/L. This assay detects TSH-binding inhibiting immunoglobulins, i.e., autoantibodies that block TSH from binding to the TSH receptor. Consequently, it measures the total TRAb titer, including both stimulating and blocking antibodies. Yearly TRAb values were collected for each patient, and missing measurements were estimated by linear interpolation [14]. Linear interpolation is a commonly used method for time-series data in which a straight line is drawn between two known data points, and the missing value is assumed to lie along this line.

We applied k-means clustering to identify distinct longitudinal patterns of TRAb changes (“Patterns A, B, C, D”) based on baseline TRAb levels, changes over time, and whether normalization occurred. In addition, we performed a cox-regression analysis using a median-based threshold of 6.14 IU/L to divide patients into “lower-TRAb” (<6.14 IU/L) and “higher-TRAb” (≥6.14 IU/L) groups. Although no formal cutoff analysis (e.g., ROC curve) was performed, 6.14 IU/L was chosen because it represented the median TRAb value in our study population.

GD treatment status during the follow-up period was collected, including the duration of methimazole prescription, statin use, RAI treatment history, and thyroidectomy. GD duration and GO comorbidities were also reviewed. GO diagnosis was confirmed by a specialized ophthalmologist at the same hospital whose primary responsibility was to manage patients with GO. The clinical parameters were compared between the different TRAb patterns and investigated to determine whether they were related to TRAb normalization.

### 2.3. Statistical Analysis

Clustering analysis: To identify homogeneous patient trajectories, we used the KmL algorithm) for functional cluster analysis. KmL, a non-parametric extension of k-means for longitudinal data, clusters based on the overall shape and temporal changes of trajectories, rather than individual points. For handling missing values, we utilized the ‘linearInterpol.local’ imputation method, which involves linear interpolation for intermittent missing values and considers the global slope at the end [17,18]. The number of clusters was determined based on clinical judgment and interpretation, including TRAb normalization rates, overall decline patterns, and time to stabilization. Given the absence of established guidelines for TRAb trajectory clustering, we empirically evaluated different configurations and selected four clusters that best represented distinct clinical patterns. Clinical parameters between the different patterns were compared using Pearson’s chi-square test and Kruskal–Wallis rank sum test.

Survival analysis: We estimated the median time to normalization of TRAb values using Kaplan–Meier curves. Survival data from the original dataset were utilized, with “survival time” defined as the duration from the index date to either normalization time or the last follow-up observation. The “status” variable was coded as 1 for normalization and 0 for cases without normalization (censored). Univariate and multivariate Cox regression analyses were conducted to identify factors affecting the time to TRAb value normalization. For the Cox models, we chose to present results as “Survival ratio” rather than the conventional hazard ratio, aiming to emphasize the proportion of patients who achieve TRAb normalization over time. Specifically, the “Survival ratio” reflects the relative rate of normalization events in one group compared to another at a given time point.

In all analyses, statistical analyses were performed using the R statistical language (version 4.0.5; R Core Team, 2021) and additional packages (kml version 2.4.1, longitudinalData version 2.4.1). A *p*-value of < 0.05 was considered statistically significant.

## 3. Results

A total of 403 patients (111 men) were included in this study. The mean age at enrollment was 47.65 ± 15.58 years, and the mean follow-up duration was 4.88 ± 2.33 years. At diagnosis, the median disease duration was 0.15 years (IQR: 0.00–0.57 years), and 63 patients (16%) presented with comorbid GO. Antithyroid medication (methimazole) was prescribed to 359 patients (89%) for an average of 44 ± 33.1 months (1320 ± 993 days). Initial dosages typically ranged from 10–30 mg/day, with gradual tapering based on biochemical response. Among the remaining 44 patients (11%) who did not receive ATD therapy, 7 patients (1.7%) underwent RAI therapy, while 37 patients (9.2%) were managed with watchful waiting due to normal thyroid function despite elevated TRAb levels. Methimazole duration varied significantly across TRAb clusters (*p* < 0.001), with the shortest duration observed in Pattern A (1118 ± 910 days) and prolonged therapy required in Patterns B (1858 ± 1078 days), C (1815 ± 944 days), and D (1792 ± 1027 days). Statin therapy was used in 40 patients (9.9%), radioactive iodine (RAI) therapy was performed in 47 (12%), and 5 (1.2%) underwent thyroidectomy during the follow-up (Table 1).

The mean baseline TRAb level was 8.98 ± 8.00 IU/L. Over a 10-year period, the mean of all measured TRAb values (“10-year average”) was 4.76 ± 6.38 IU/L, with a within-patient standard deviation (“10-year SD”) of 4.10 ± 3.74 IU/L. The annual rate of TRAb change was −0.29 ± 1.26 IU/L/year, indicating a general decline in TRAb levels across most patients, although some exhibited stable or even increasing trends. In total, 333 patients (83%) achieved TRAb normalization (<1.5 IU/L), and the mean time to normalization was 2.60 ± 1.87 years.

### 3.1. Patterns of TRAb over Time and Overall Clinical Information

**Figure 1:**
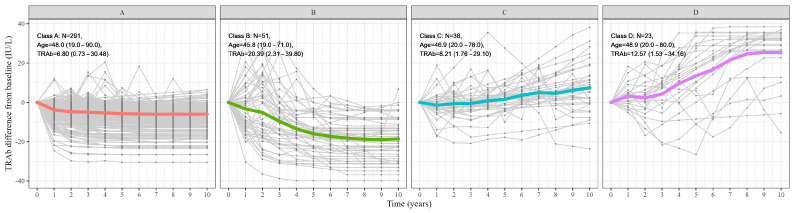
Four Patterns of Long-Term TRAb Changes in Graves’ Disease. Four distinct patterns of thyroid-stimulating hormone receptor antibody (TRAb) change over time in Graves’ disease: (**A**–**D**). Each pattern demonstrated a different baseline, rate of change, and normalization rate of TRAb. TRAb, thyroid-stimulating hormone receptor antibody.

Out of 4433 total TRAb data points, 567 interpolated data points were generated after excluding measurements obtained following TRAb normalization (with no subsequent follow-up). Four distinct patterns of TRAb were identified over time and were designated as A, B, C, and D based on initial TRAb levels, annual rates of change, and final normalization outcomes (Figure 1, Table 1).

Pattern A (n = 291, 72.2%) had the lowest baseline TRAb (6.8 ± 5.42 IU/L) and showed a mean TRAb change of −0.41 ± 0.41 IU/L/year. This group attained a 96% normalization rate, with a median survival time of 2 years (*p* < 0.001).

Pattern B (n = 51, 12.6%) had the highest baseline TRAb (20.39 ± 10.21 IU/L) and exhibited a steep negative trend (−1.98 ± 0.77 IU/L/year). TRAb normalization occurred in 80% of these patients, and the median survival time was 8 years (*p* < 0.001).

By contrast, Patterns C (n = 38, 9.4%) and D (n = 23, 5.7%) showed positive mean TRAb changes (0.88 ± 1.28 IU/L/year and 2.95 ± 1.65 IU/L/year, respectively) and lower normalization rates (29% and 13%). Fewer than half of the patients in Patterns C and D achieved normalization within the study period (*p* < 0.001). Patterns B and D demonstrated steeper annual TRAb changes than A and C (*p* < 0.001), and Patterns A and B had higher final normalization rates (96% and 80%) than C and D (29% and 13%) (*p* < 0.001).

Pattern D exhibited the most refractory disease course, characterized by persistently high and increasing TRAb levels over time. Despite prolonged ATD therapy with a mean duration of 4.9 years, Pattern D had the lowest TRAb normalization rate (13%), indicating that most patients failed to achieve immunologic remission. Compared to other patterns, Pattern D patients had higher rates of radioactive iodine therapy (30%) and highest thyroidectomy (8.7%, *p* = 0.006), suggesting that definitive treatment was frequently required. However, even with these interventions, many Pattern D patients remained TRAb-positive post-treatment, indicating persistent autoimmune activity.

Among these four patterns, Pattern B had the longest mean disease duration (1.02 ± 1.48 years, *p* = 0.018). Comorbidity with GO did not significantly differ across patterns (*p* = 0.876). MMI was prescribed for the shortest duration in Pattern A (1118 ± 910 days, *p* < 0.001), while RAI therapy was more frequently used in Patterns B (35%), C (16%), and D (30%) than in Pattern A (5.5%, *p* < 0.001). Statin prescription rates did not differ significantly among the four groups (*p* = 0.759). Detailed demographic and clinical variables for each pattern are provided in Table 1.

### 3.2. Two Main Patterns Based on Normalization Rate

When Pattern A was compared with the combined Patterns B, C, and D (hereafter “BCD group”), Pattern A showed a significantly higher normalization rate. Specifically, 278 of 291 patients (96%) in Pattern A achieved TRAb normalization, whereas only 55 of 112 patients (49%) in the BCD group did so (*p* < 0.001). Consistent with these findings, the baseline TRAb level in Pattern A was markedly lower (6.80 ± 5.42 IU/L) than in the BCD group (14.65 ± 10.50 IU/L; *p* < 0.001; Table 2).

Disease duration was also shorter in Pattern A (0.13 years, IQR: 0.00–0.51 years) compared with the BCD group (0.23 years IQR: 0.03–1.03 years; *p* = 0.004). Methimazole (MMI) therapy was prescribed for an average of 1118 ± 910 days in Pattern A, significantly less than the 1830 ± 1013 days in the BCD group (*p* < 0.001). In addition, the BCD group had higher rates of radioactive iodine (RAI) therapy (28% vs. 5.5%, *p* < 0.001) and thyroidectomy (3.6% vs. 0.3%, *p* = 0.022). No significant differences in MMI usage frequency (*p* = 0.427) or statin prescription (*p* = 0.246) were noted between the two groups.

The mean TRAb change rate was −0.41 ± 0.41 IU/L/year in Pattern A, compared with 0.00 ± 2.28 IU/L/year in the BCD group (*p* < 0.001). Likewise, the time to TRAb normalization was shorter in Pattern A (2.39 ± 1.64 years) than in the BCD group (4.27 ± 2.64 years; *p* < 0.001). Detailed demographic and clinical variables for both Pattern A and the BCD group are summarized in Table 2.

### 3.3. Survival Analysis of TRAb

**Figure 2:**
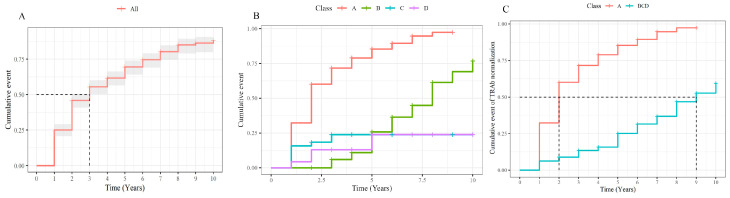
Kaplan-Meier Curves of TRAb Normalization Across Overall Patients and Patterns in Graves’ Disease. (**A**) Figure 2A depicts the Kaplan–Meier survival curve, demonstrating the time to thyroid-stimulating hormone receptor antibody (TRAb) normalization among the total patients over a 10-year follow-up period. The median time of normalization was observed to be 3 years, by the end of the 10-year period, approximately 80% of the patients had achieved TRAb normalization. (**B**) Kaplan–Meier curve of TRAb normalization in each of the four TRAb change patterns. A and B achieved a high normalization rate compared to C and D. A revealed a faster TRAb normalization compared to B while C showed a faster TRAb normalization compared to D. (**C**) Kaplan–Meier curve of TRAb normalization in A and BCD patterns. A showed a higher and faster normalization pattern compared to BCD. TRAb, thyroid-stimulating hormone receptor antibody. The black dashed line indicates the time point at which 50% of patients achieved TRAb normalization.

The median time to TRAb normalization in the overall cohort was three years (Figure 2A). When patients were classified into the four predefined patterns (A, B, C, D), Pattern A had a median survival time of two years, whereas Pattern B showed a notably longer median survival time of eight years. In Patterns C and D, fewer than half of the patients achieved normalization within the 10-year observation period.

An additional survival analysis compared Pattern A with the BCD group. In this comparison, the BCD group demonstrated a median survival time of nine years (Figure 2B,C), indicating a slower normalization rate than that observed in Pattern A.

### 3.4. Prognostic Factors Associated with Patterns

We performed survival analyses using a Cox regression model but present the results as “Survival ratio” (SR) rather than the conventional hazard ratio. These analyses demonstrated that Pattern A was significantly associated with a higher probability of TRAb normalization compared with Patterns B, C, and D (*p* < 0.001; Table 3). In the multivariable model, the SR for TRAb normalization in Pattern A was 8.391 (95% CI: 3.087–22.81), whereas Patterns B and C did not show significant differences relative to Pattern D.

Among the patient characteristics examined, female sex increased the likelihood of TRAb normalization by approximately 1.3-fold (*p* = 0.044), and having an initial TRAb level below 6.14 IU/L was associated with a 1.7-fold higher probability of normalization (*p* < 0.001). Age, presence of GO, methimazole (MMI) treatment, statin use, and radioactive iodine (RAI) therapy were not significant predictors of TRAb normalization in the multivariable analysis. Although RAI therapy showed a reduced likelihood of TRAb normalization in the univariate model (SR, 0.415; 95% CI: 0.272–0.632; *p* < 0.001), this effect did not persist in the multivariable model (SR, 0.715; 95% CI: 0.459–1.113; *p* = 0.138). Thyroidectomy also did not exhibit a statistically significant association with TRAb normalization in the final analysis.

In a separate comparison of Pattern A (Group A) versus the combined BCD group (Patterns B, C, and D), similar results were obtained: female sex and lower baseline TRAb (<6.14 IU/L) were significantly linked to faster TRAb normalization. Detailed SRs and confidence intervals for each factor are provided in Table 3.

## 4. Discussion

Our findings demonstrate that the median time to TRAb normalization in this cohort was three years, underscoring the heterogeneous clinical courses of GD. Based on baseline TRAb levels, rate of changes, and normalization rates, we identified four distinct patterns. These could be further grouped into two main categories depending on whether the median time to normalization was within two years. Pattern A rapidly achieved normalization (median two years), whereas the remaining patterns generally required longer durations or exhibited normalization rates below 50%. Although these discrepancies were linked to factors such as disease duration, treatment modalities, and baseline TRAb concentrations, GO comorbidity rates did not differ significantly across the patterns. Notably, female sex and lower initial TRAb (<6.14 IU/L) were associated with greater probabilities of achieving normalization.

Among these patterns, Pattern A—observed in the majority of our cohort—appears to reflect the most favorable disease trajectory. Patients in Pattern A had the lowest baseline TRAb, the shortest time to normalization, less frequent use of RAI, and shorter durations of MMI therapy. This aligns with earlier reports indicating that an early and sustained response to a 12–18-month course of MMI often leads to remission in GD [6,19]. Patterns B, C, and D demonstrated relatively higher initial TRAb levels, longer methimazole prescription durations, and higher RAI and thyroidectomy prescription rates than Pattern A. While methimazole was prescribed at similar frequencies across clusters, the duration of therapy was significantly longer in Patterns B, C, and D, suggesting a need for prolonged ATD management in patients with persistently elevated TRAb levels. Notably, a high baseline TRAb level is a recognized risk factor for relapse [20,21], and discontinuation of antithyroid medication has been associated with relapse rates of 40–50% in Europe and Japan and approximately 37% in South Korea [22,23]. Consistent with these observations, the 2016 American Thyroid Association guidelines recommend either extending antithyroid therapy or opting for definitive treatments (RAI or surgery) in patients who demonstrate persistently elevated TRAb levels or experience clinical relapse [6].

The relatively low number of patients who underwent RAI therapy or thyroidectomy in our cohort can be attributed to both study design and institutional treatment preferences. At our center, long-term antithyroid drug (ATD) therapy is generally preferred over definitive treatments, aligning with broader clinical practice trends in South Korea. Extended ATD therapy has been associated with improved remission rates, reducing the need for early definitive interventions. Additionally, concerns regarding RAI-induced exacerbation of Graves’ orbitopathy (GO) contribute to this treatment approach. Thyroidectomy is typically reserved for cases of refractory hyperthyroidism, large goiters causing compressive symptoms, suspected or confirmed thyroid malignancies, contraindications to RAI, or patient preference. These factors likely contributed to the lower proportion of RAI and thyroidectomy cases observed in our cohort.

Patterns B, C, and D likely represent clinical trajectories in which patients failed to achieve remission with an initial MMI course or experienced relapse after medication withdrawal. While Patterns B, C, and D had smaller sample sizes compared to Pattern A, their TRAb trajectories exhibited well-defined and reproducible trends, reinforcing their clinical relevance. Nevertheless, to further enhance statistical reliability and confirm the robustness of these clusters, future studies will be essential. Expanding the dataset will help validate these TRAb trajectory classifications and refine our understanding of long-term disease progression. These patterns showed higher baseline TRAb levels, more frequent second-line or ablative interventions, and extended MMI prescriptions, consistent with earlier reports that prolonged or repeated antithyroid therapy—combined with elevated TRAb—often necessitates RAI or thyroidectomy [23,24]. Notably, Pattern B achieved a higher rate of TRAb normalization (80%) than Patterns C (29%) and D (13%), despite exhibiting the highest baseline TRAb level. Although Pattern B required a longer median time (eight years) to normalization than Pattern A, its eventual success highlights the role of additional or extended therapies for patients who fail to achieve remission after the initial MMI course. Indeed, previous studies have noted that a substantial proportion of GD patients who relapse following first-line MMI can still attain euthyroidism with prolonged medical therapy or RAI [25]. Still, the median survival time of 8 years and a mean TRAb change rate of −1.98 ± 0.77 IU/L/year in Pattern B highlight the impact of baseline TRAb levels on long-term outcomes [26]. Even with comprehensive management—including pharmacological and surgical interventions—achieving TRAb normalization within a widely acceptable timeframe (e.g., 2–3 years) can remain elusive for many patients [23].

Patterns C and D further illustrate the complexity of TRAb dynamics by offering distinct pathways to persistent elevation. Pattern C began with a relatively elevated baseline TRAb but exhibited only a modest positive slope, resulting in a 29% normalization rate. In contrast, Pattern D, despite starting from a moderate baseline TRAb, showed both significant fluctuations and an overall upward trend, even after multiple treatments, including RAI and, in 8.7% of cases, thyroidectomy. These fluctuations may reflect ongoing immune dysregulation, where TRAb levels remain unstable due to persistent autoantibody activity. While TRAb fluctuations have not been directly correlated with worse prognosis or treatment response, their presence may indicate an unstable disease course, potentially requiring more intensive monitoring and individualized therapeutic strategies. Some individuals in Pattern D maintained persistently elevated TRAb levels following surgery, potentially reflecting residual thyroid tissue or ongoing autoimmune activity [27]. The presence of persistent fluctuations, rather than a steady increase, suggests that antibody production remains dysregulated, possibly influenced by genetic predisposition, immune-modulating factors, or incomplete suppression of thyroid autoimmunity. This resistance to conventional treatments underscores the need for more aggressive or alternative therapeutic approaches and suggests possible immunologic or genetic underpinnings [5,28,29,30].

Regarding GO, our data showed no significant difference in GO comorbidity across these TRAb patterns, despite notable variations in RAI use. Specifically, 28% of patients in the combined BCD group underwent RAI compared with 14% in Pattern A (*p* < 0.001), yet GO incidence was 14% vs. 16%, respectively. This may be partly due to the concurrent and prolonged use of MMI in nearly 90% of the BCD group. In line with these findings, Azizi et al. [31] demonstrated that concurrent MMI administration reduces the risk of GO exacerbation in patients receiving RAI. Nevertheless, prospective studies are warranted to clarify whether extended antithyroid therapy truly mitigates GO risk in individuals presenting with high baseline TRAb. This is because others report that the impact of MMI preventing GO risks is minimal and maintaining euthyroidism and concurrent steroid treatment are more critical factor [25]. In addition to MMI use, several confounding factors likely influenced GO incidence across TRAb patterns. Prophylactic corticosteroid administration during RAI therapy has been shown to significantly reduce the risk of GO worsening [12,26]. However, data on corticosteroid use in our RAI-treated patients were not systematically collected, so we could not assess how many individuals received steroid prophylaxis. Given that steroid prophylaxis is often recommended for high-risk patients, it is possible that some of our RAI-treated patients received this intervention, which could have mitigated GO exacerbation. This potential protective effect should be considered when interpreting the lack of GO incidence differences across TRAb patterns.

Although our study did not find significant differences in GO prevalence among TRAb trajectory groups, high TRAb remains a well-established risk factor for GO development and progression. Given this, regular TRAb measurement every 6–12 months is recommended, particularly in high-risk individuals. Furthermore, patients with an initial TRAb level ≥6.14 IU/L frequently exhibited persistently elevated levels, suggesting that this subgroup may require closer monitoring for potential GO progression or disease relapse.

The observed GO comorbidity rates of 11–17% are lower than the 27% reported by Tanda et al. [32], potentially reflecting differences in study design and patient selection. We required at least three years of TRAb follow-up, which may have excluded some patients with severe GO who lacked the requisite longitudinal data or were referred early for specialized care. Although no significant differences in GO comorbidities emerged among the four patterns, other investigations have found that higher TRAb levels correlate with more severe GO [10]. Future work examining GO severity rather than mere presence or absence may provide deeper insights.

Our results also corroborate previous evidence that female sex and lower initial TRAb levels predict a higher likelihood of TRAb normalization. Women have been reported to achieve higher remission rates than men [33], and an elevated baseline TRAb is a well-established marker for relapse. Cappelli et al. [27] similarly demonstrated that TRAb titers at GD diagnosis can help forecast long-term outcomes, where a high titer predicts persistent disease.

Despite identifying four distinct patterns of TRAb changes, clinicians should note that significant variability can exist within each cluster. Although these patterns offer a valuable framework for understanding general trajectories, individual patient courses may deviate substantially from the cluster average. Such heterogeneity reflects the multifactorial nature of GD, encompassing genetic predispositions, immune-regulatory differences, and variable treatment responses [27]. Accordingly, these patterns should be treated as broad guides rather than definitive predictions of each patient’s course, reinforcing the need for personalized approaches, possibly incorporating additional biomarkers and clinical variables.

Our clustering approach utilized imputation-based K-means clustering, where missing data were handled using imputation techniques before analysis. The number of clusters (four) was selected empirically, considering TRAb normalization rates and decline trends, as no established guidelines exist for TRAb-based clustering. We tested multiple configurations (three to six clusters) and found that four clusters provided the most meaningful differentiation without excessive fragmentation. However, this selection was based on clinical interpretation rather than formal statistical validation metrics. Future studies should incorporate quantitative validation techniques to refine cluster determination and ensure robustness across different patient populations.

Our study has several limitations. First, its retrospective and single-center design may introduce biases, particularly in assessing GO comorbidities, as not all GD patients underwent ophthalmological evaluation. Second, we did not classify relapse vs. remission in our TRAb analysis, which may overlook certain clinical nuances. Third, while linear interpolation helped manage missing data, it may have obscured short-term TRAb fluctuations with potential prognostic value.

Additionally, some relevant clinical parameters were not systematically recorded in our dataset, which limits the scope of our analysis. Specifically, smoking status, MRI findings (extraocular muscle involvement), biological therapy usage, proptosis severity, therapy compliance, and serial TSH4/TSH3 levels were unavailable. These factors may influence disease outcomes, particularly in refractory cases such as Pattern D. Future prospective studies incorporating these variables would provide a more comprehensive understanding of the clinical trajectory of TRAb patterns.

Furthermore, selection bias could have affected our findings, as our inclusion criteria required ≥3 years of TRAb follow-up, potentially excluding patients who underwent early thyroidectomy or RAI, leading to an underrepresentation of severe cases with distinct TRAb trajectories. Moreover, thyroidectomy patients are often followed in surgical departments rather than endocrinology, which may have contributed to the lower observed thyroidectomy rates in our cohort. Future studies should include a more diverse patient population, particularly those undergoing early definitive therapy, to refine our understanding of TRAb trajectories in different treatment scenarios.

In conclusion, we identified four distinct TRAb patterns in GD, which could be consolidated into two broader groups based on median survival times for normalization. Each pattern reflects varying TRAb trajectories and therapeutic intensities, offering clinicians a potential tool to anticipate disease courses and customize treatment strategies. Prospective, multicenter studies that incorporate a broader range of clinical, immunologic, and imaging parameters are necessary to validate these findings and further elucidate the interplay between TRAb patterns, GO severity, and long-term remission rates.

## Figures and Tables

**Table 1:** Demographics in the overall study population and four distinct patterns of TRAb change over time in Graves’ disease.

	Overall	A	B	C	D	*p*-Value *
N (%)	403	291 (72.2)	51 (12.6)	38 (9.4)	23 (5.7)	
Follow up duration (year)	4.88 (2.33)	4.68 (2.30)	5.84 (2.44)	5.21 (2.18)	4.74 (2.12)	0.006
Age (year)	47.65 (15.58)	47.96 (15.10)	45.80 (15.49)	46.95 (17.92)	48.96 (18.25)	0.837
Sex (male:female)	111:292	78:213	11:40	13:25	9:14	0.336
Disease duration (years), Median (IQR)	0.15 (0.00–0.57)	0.13 (0.00–0.51)	0.35 (0.05–1.44)	0.23 (0.02–0.54)	0.22 (0.02–0.95)	0.018
Graves’ orbitopathy comorbidity (N (%))	63 (16%)	47 (16%)	8 (16%)	4 (11%)	4 (17%)	0.876
Methimazole	359 (89%)	257 (88%)	46 (90%)	36 (95%)	20 (87%)	0.697
Methimazole prescription (day)	1320 (993)	1118 (910)	1858 (1078)	1815 (944)	1792 (1027)	<0.001
Statin	40 (9.9%)	32 (11%)	4 (7.8%)	2 (5.3%)	2 (8.7%)	0.759
Radioactive iodine treatment	47 (12%)	16 (5.5%)	18 (35%)	6 (16%)	7 (30%)	<0.001
Thyroidectomy	5 (1.2%)	1 (0.3%)	2 (3.9%)	0 (0%)	2 (8.7%)	0.006
Baseline TRAb (IU/L)	8.98 (8.00)	6.80 (5.42)	20.39 (10.21)	8.21 (6.36)	12.57 (9.93)	<0.001
10-year average of TRAb (IU/L)	4.76 (6.38)	1.81 (1.36)	7.72 (3.05)	10.61 (3.47)	25.88 (4.12)	<0.001
10-year SD of TRAb (IU/L)	4.10 (3.74)	2.36 (1.74)	8.86 (2.49)	6.00 (3.44)	12.36 (3.40)	<0.001
Change rate of TRAb (IU/L/year)	−0.29 (1.26)	−0.41 (0.41)	−1.98 (0.77)	0.88 (1.28)	2.95 (1.65)	<0.001
Normalization rate of TRAb (N (%))	333 (83%)	278 (96%)	41 (80%)	11 (29%)	3 (13%)	<0.001
Period to normalization (year)	2.60 (1.87)	2.39 (1.64)	5.85 (2.03)	1.56 (0.88)	2.50 (1.73)	<0.001

Values are presented as means (standard deviations) or numbers (%). TRAb, thyroid-stimulating hormone receptor antibody; SD, standard deviation. * Pearson’s chi-square test, Kruskal–Wallis rank-sum test, or Fisher’s exact test.

**Table 2:** Demographics in the overall study population and two re-clustered patterns (A vs. BCD) of TRAb change over time in Graves’ disease.

	Overall	A	BCD	*p*-Value *
N (%)	403	291 (72.2)	112 (27.7)	
Follow up duration (year)	4.88 (2.33)	4.68 (2.30)	5.40 (2.32)	0.002
Age (year)	47.65 (15.58)	47.96 (15.10)	46.84 (16.81)	0.592
Sex (male:female)	111:292	78:213	33:79	0.592
Disease duration (years), Median (IQR)	0.15 (0.00–0.57)	0.13 (0.00–0.51)	0.23 (0.03–1.03)	0.004
Graves’ orbitopathy comorbidity (N (%))	63 (16%)	47 (16%)	16 (14%)	0.644
Methimazole	359 (89%)	257 (88%)	102 (91%)	0.427
Methimazole prescription duration (day)	1320 (993)	1118 (910)	1830 (1013)	<0.001
Statin	40 (9.9%)	32 (11%)	8 (7.1%)	0.246
Radioactive iodine treatment	47 (12%)	16 (5.5%)	31 (28%)	<0.001
Thyroidectomy	5 (1.2%)	1 (0.3%)	4 (3.6%)	0.022
Baseline TRAb (IU/L)	8.98 (8.00)	6.80 (5.42)	14.65 (10.50)	<0.001
10-year average of TRAb (IU/L)	4.76 (6.38)	1.81 (1.36)	12.43 (7.77)	<0.001
10-year SD of TRAb (IU/L)	4.10 (3.74)	2.36 (1.74)	8.61 (3.78)	<0.001
Change rate of TRAb (IU/L/year)	−0.29 (1.26)	−0.41 (0.41)	0.00 (2.28)	<0.001
Normalization rate of TRAb (N (%))	333 (83%)	278 (96%)	55 (49%)	<0.001
Period to normalization (year)	2.60 (1.87)	2.39 (1.64)	4.27 (2.64)	<0.001

Values are presented as means (standard deviations) or numbers (%). TRAb, thyroid-stimulating hormone receptor antibody; SD, standard deviation. * Pearson’s chi-square test, Kruskal–Wallis rank-sum test, or Fisher’s exact test.

**Table 3:** Survival analysis using Cox model to examine factors related to normalization of thyroid-stimulating hormone receptor antibody (TRAb).

	Univariate Analysis			Multivariable Analysis		
Characteristic	Survival Ratio	95% Confidence Interval	*p*-Value	Survival Ratio	95% Confidence Interval	*p*-Value
TRAb pattern						
*D*	1	Ref.		—	—	
*A*	8.873	3.285, 23.97	**<0.001**	8.391	3.087, 22.81	**<0.001**
*B*	1.860	0.635, 5.445	0.258	1.858	0.632, 5.461	0.260
*C*	1.422	0.437, 4.626	0.559	1.218	0.373, 3.981	0.744
Sex						
M	1	Ref.		—	—	
F	1.227	0.942, 1.598	0.129	1.317	1.007, 1.723	**0.044**
Age (year)						
≥19 and <49	1	Ref.		—	—	
≥49	1.065	0.846, 1.341	0.590	1.096	0.864, 1.391	0.449
GO comorbidity						
No	1	Ref.		—	—	
Yes	1.056	0.760, 1.466	0.747	1.135	0.805, 1.601	0.470
Methimazole						
No	1	Ref.		—	—	
Yes	0.866	0.608, 1.233	0.424	1.096	0.763, 1.575	0.618
Statin						
No	1	Ref.		—	—	
Yes	1.188	0.823, 1.715	0.358	1.1	0.747, 1.620	0.630
RAI treatment						
No	1	Ref.		—	—	
Yes	0.415	0.272, 0.632	<0.001	0.715	0.459, 1.113	0.138
Thyroidectomy						
No	1	Ref.		—	—	
Yes	1.896	0.266, 13.53	0.523	—	—	
Baseline TRAb (IU/L)						
≥15 and <6.14	1	Ref.		—	—	
≥6.14	2.104	1.660, 2.666	**<0.001**	1.746	1.363, 2.235	**<0.001**

TRAb, thyroid-stimulating hormone receptor antibody; GO, Graves’ orbitopathy; RAI, radioactive iodine. *p*-value in bold indicates significance at *p* = 0.05.

## Data Availability

Data can be provided upon request.
